# Dietary Cocoa Powder Improves Hyperlipidemia and Reduces Atherosclerosis in apoE Deficient Mice through the Inhibition of Hepatic Endoplasmic Reticulum Stress

**DOI:** 10.1155/2016/1937572

**Published:** 2016-02-15

**Authors:** Hua Guan, Yan Lin, Liang Bai, Yingfeng An, Jianan Shang, Zhao Wang, Sihai Zhao, Jianglin Fan, Enqi Liu

**Affiliations:** ^1^Research Institute of Atherosclerotic Disease, Xi'an Jiaotong University Cardiovascular Research Center, Xi'an, Shaanxi 710061, China; ^2^Laboratory Animal Center, Xi'an Jiaotong University Health Science Center, Xi'an, Shaanxi 710061, China; ^3^Department of Molecular Pathology, Interdisciplinary Graduate School of Medicine and Engineering, University of Yamanashi, Yamanashi 409-3898, Japan

## Abstract

Cocoa powder is rich in flavonoids, which have many beneficial effects on human health, including antioxidative and anti-inflammatory effects. The aim of our study was to investigate whether the intake of cocoa powder has any influence on hyperlipidemia and atherosclerosis and examine the underlying molecular mechanisms. We fed apoE knockout mice a Western diet supplemented with either 0.2% (low group) or 2% (high group) cocoa powder for 12 weeks. The groups fed dietary cocoa powder showed a significant reduction in both plasma cholesterol levels and aortic atherosclerosis compared to the control group. Analysis of mRNA profiling of aortic atherosclerotic lesions revealed that the expression of several genes related to apoptosis, lipid metabolism, and inflammation was significantly reduced, while the antiapoptotic gene Bcl2 was significantly increased in the cocoa powder group compared to the control. RT-PCR analysis along with Western blotting revealed that a diet containing cocoa powder inhibited the expression of hepatic endoplasmic reticulum stress. These data suggest that cocoa powder intake improves hyperlipidemia and atherosclerosis, and such beneficial effects are possibly mediated through the suppression of hepatic endoplasmic reticulum stress.

## 1. Introduction

Cocoa powder is a food consumed in many countries throughout the world [1]. Cocoa powder contains high amounts of flavonoids, a class of plant and fungus secondary metabolites, which are considered to have beneficial effects on human health. Historically, the Olmec, Mayan, and Aztec people used cocoa powder to treat various types of diseases [2, 3]. It has been demonstrated that cocoa powder has a number of physiological effects, such as antioxidant effects, anti-inflammatory effects, and improvement of endothelial cell functions, which improve cardiovascular functions [4, 5].

Previous studies demonstrated that feeding animals with cocoa liquor polyphenols inhibited low density lipoprotein (LDL) oxidation in rabbits and prevented blood glucose elevation in diabetic obese mice [6, 7]. In addition, treatment with cocoa phenolic extracts protected pancreatic beta cells against oxidative stress [8]. Cocoa powder has been shown to have other beneficial effects on immune disease [9], cancers [10], oxidative injuries [11], inflammatory conditions [12–14], hyperglycemia [15], and insulin resistance [16]. Based on these findings, we envisioned that cocoa powder may have effects on hyperlipidemia and atherosclerosis. To test this hypothesis, we fed apoE knockout (KO) mice, the most popular animal model for human hyperlipidemia and atherosclerosis, with dietary cocoa powder for 12 weeks and compared their lipid profiles, aortic atherosclerosis, and hepatic mRNA expression with those of the control mice. We attempted to answer two questions in the current study: (1) Does dietary supplementation with cocoa powder affect plasma lipids and aortic atherosclerosis? (2) If so, what is the molecular mechanism? Our results demonstrated that a diet with cocoa powder significantly improved hyperlipidemia and inhibited aortic atherosclerosis.

## 2. Materials and Methods

### 2.1. Animals and Diets

Male apoE KO mice were provided by the Laboratory Animal Center of Xi'an Jiaotong University at the age of 8 weeks. Cocoa beans were purchased from China General Technology Holding Co., Ltd. (Beijing, China) and ground into cocoa powder. The compositions of cocoa powder are shown in Supplementary Table  1 of the Supplementary Material available online at http://dx.doi.org/10.1155/2016/1937572. Cocoa powder groups were fed a Western diet containing 0.15% cholesterol and 21% fat, which was supplemented with either 0.2% (low) or 2% (high) cocoa powder. The control group was fed a Western diet alone. The detailed mouse Western diet compositions are listed in Supplementary Table  2. Each group was composed of fifteen animals. The mouse diets were made by Vital River Company (Vital River Company, Beijing, China). All mice were housed in an air-conditioned room under a 12 h light and 12 h dark cycle. Feed and water were allowed* ad libitum*. The animal experiment protocol was approved by the Laboratory Animal Administration Committee of Xi'an Jiaotong University and carried out according to the Guidelines for Animal Experimentation of Xi'an Jiaotong University and Guide for the Care and Use of Laboratory Animals published by the US National Institutes of Health (NIH Publication number 85-23, revised 2011).

### 2.2. Biochemical Analyses

Blood was collected via the tail vein after mice were fasted overnight. Blood in the tubes with EDTA was centrifuged (1,500 rpm for 10 min at 4°C) to obtain plasma. Plasma total cholesterol (TC), LDL cholesterol (LDL-C), and high density lipoprotein cholesterol (HDL-C) were analyzed using commercial assay kits (Biosino Bio-Technology & Science Inc., Beijing, China) [17].

To evaluate plasma oxidative stress, we measured the superoxidase dismutase activity, malonyldialdehyde, total nitric oxide synthase, glutathione peroxidase, glutathione *s*-transferase, total antioxidant capacity, and xanthine oxidase (XOD) activity with colorimetric assay kits. Nitric oxide was measured using a nitrate reductase assay kit. All assay kits were purchased from Nanjing Jiancheng Bioengineering Institute (Nanjing, China).

All plasma samples were from individual animal and each sample was analyzed in triplicate and measured according to manufacturer's protocol using a Benchmark microplate reader (170-6750XTU, Bio-Rad, Veenendaal, Netherlands).

### 2.3. Atherosclerotic Lesion Quantification

For quantification of atherosclerosis, animals were euthanized by intraperitoneal pentobarbital injection (100 mg/kg) and aortic trees were dissected and stained with oil red O. The* en face* lesion size was analyzed with the image analysis system (WinRoof Mitani Co., Tokyo, Japan) [18].

For analysis of microscopic areas of atherosclerotic lesions, frozen cross sections were cut at the level of the aortic root. Ten cross sections were analyzed from each mouse. To quantify the lesion area, the sections were stained with hematoxylin-eosin (H&E) and oil red O. The area stained by oil red O staining was quantified [19].

### 2.4. PCR Array Analysis

To evaluate changes in gene expression in the atherosclerotic lesions, mRNA levels of aortic lesions were compared using a PCR array analysis. Total RNA from aortas was isolated using an RNeasy Fibrous Tissue Mini Kit (Qiagen, Germantown, MD, USA) according to the manufacturer's instructions; then, they were reversely transcribed using a Maxima First Strand cDNA Synthesis Kit (Qiagen). We performed PCR array analysis using a mouse atherosclerosis PCR array kit (Qiagen). All plates had positive controls and reverse transcription controls. Values of the cycle threshold (Ct) obtained for quantification were used for calculations of fold-change in mRNA abundance following the 2^−ΔΔCt^ method. *β*-actin was chosen as the housekeeping gene [20]. The results of the PCR array are shown in Supplementary Table  3.

### 2.5. Real Time PCR Analysis

Total RNA from mouse livers was extracted using TRIzol Plus (Invitrogen, Carlsbad, CA, USA), and cDNA was synthesized with a SuperScript® III First-Strand Synthesis System (Invitrogen, Carlsbad, CA, USA). Real time PCR analysis was performed using TaKaRa TP800 (TaKaRa Biology Inc., Shiga, Japan). The number of transcripts was quantified, and each sample was normalized according to its *β*-actin content. PCR primer sequences are shown in Supplementary Table  4.

### 2.6. Western Blotting Analysis

Fresh liver samples were homogenized in lysis buffer at 4°C followed by centrifugation at 12,000 rpm at 4°C for 10 min to remove debris. The resultant supernatants were collected and subjected to Western blotting. Briefly, 20 *μ*g lysates were fractionated on 10% SDS-polyacrylamide gels and then transferred to Sequi-Blot polyvinylidene fluoride membranes (Bio-Rad, Hercules, CA, USA). The membranes were incubated with each primary antibody (Ab) (anti-XBP1 1 : 1000, anti-BiP 1 : 1000, anti-p-PERK 1 : 1000, and *β*-actin 1 : 1000) at 4°C overnight, as recommended in the manufacturer's instructions. After washing 3 times, they were incubated with horseradish peroxidase-conjugated secondary Ab for 2 hr. Signals were detected using the Immobilon reagent (Millipore, Billerica, MA, USA) and visualized using an LAS-400 Lumino Image Analyzer (Fujifilm, Co., Tokyo). Visualized signal intensities were quantitatively analyzed using MultiGauge software (Bio-Rad, Hercules, CA, USA). All primary Abs were purchased from Cell Signaling Technology (Beverly, MA, USA).

### 2.7. Analysis of Hepatic Lipids

100 mg fresh liver sample was homogenized with 1.9 mL chloroform-methanol (2 : 1) mixture (v/v) to a final dilution volume of the liver sample; that is, the liver homogenate from 0.1 g of tissue was diluted to a volume of 2 mL. The homogenate was extracted overnight (4°C) and centrifuged (3000 rpm, 20 min). Pipetted 100 *μ*L liquids from the lower phase contained essentially all the tissue lipids and were transferred into a glass tube. These lipids were dried with nitrogen to make chloroform diffusion and all of the lipids almost remained in the bottom of the tube. The lipids were dissolved with 50 mL ethanol and these were used to measure liver TC and triglyceride (TG) using commercial assay kits (Biosino Bio-Technology & Science Inc., Beijing, China) [21].

### 2.8. Statistical Analysis

All data are expressed as the mean ± SEM. Statistical analyses were performed using either Student's *t*-test with an equal *F* value or Welch's *t*-test when the *F* value was not equal. We performed Student's *t*-test to analyze PCR array results (control group versus high dose cocoa powder group). Three-group data were analyzed by One-Way ANOVA followed by Tukey's multiple-comparison procedure. For unequal variances, data were evaluated by Kruskal-Wallis test. Statistical significance was determined to be reached at a *P* value of less than 0.05.

## 3. Results

### 3.1. Plasma Parameters

**Figure 1:**
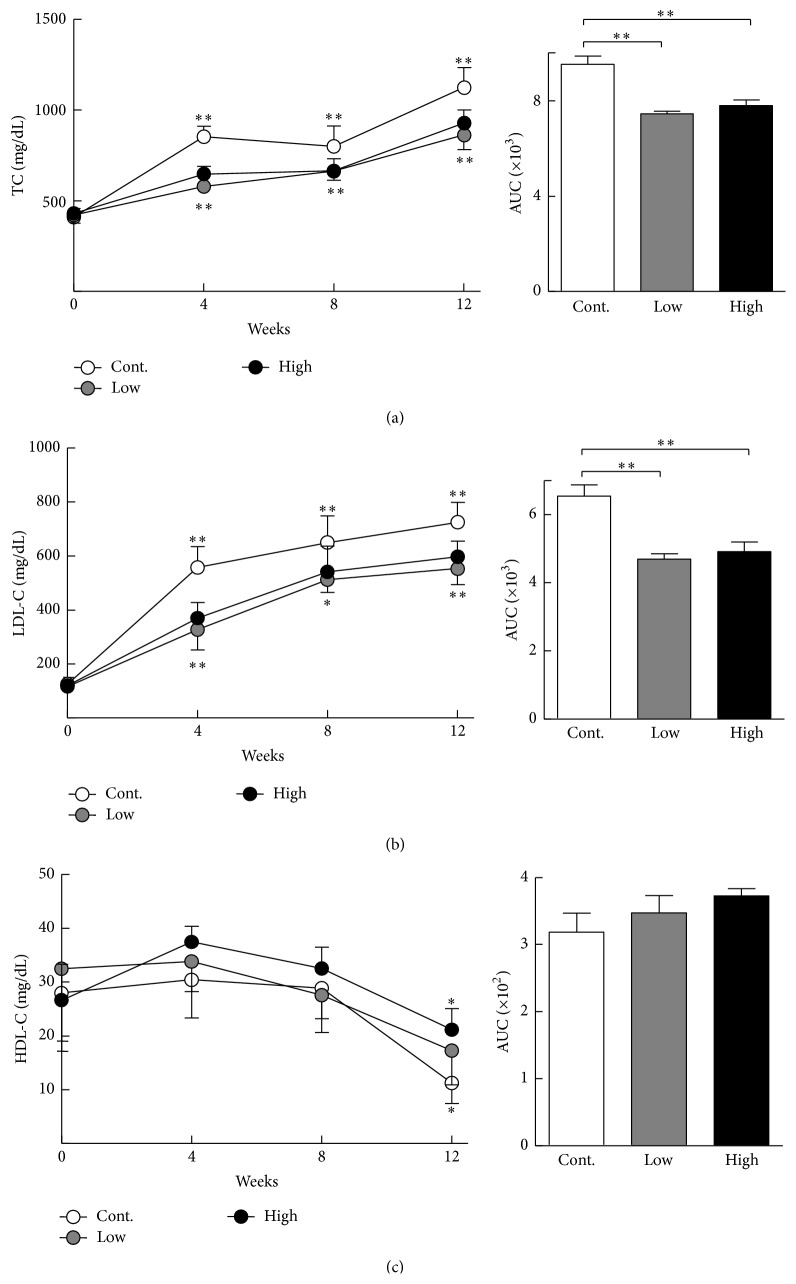
Plasma levels of total cholesterol (TC), low density lipoprotein cholesterol (LDL-C), and high density lipoprotein cholesterol (HDL-C). Data are expressed as the mean ± SEM. *n* = 15 for each group. ^*∗*^ *P* < 0.05, ^*∗∗*^ *P* < 0.01 versus control.

Cocoa power feeding (at both low and high doses) reduced the plasma levels of TC at 4, 8, and 12 weeks compared with the control. When plasma TC levels were expressed by area under the curve (AUC), the statistical significance was more evident: there was a 21% reduction in the low group and an 18% reduction in the high group (Figure 1). Reduced plasma TC levels were attributed to reduction of plasma LDL-C in the cocoa powder groups (i.e., 28% reduction in low group and 25% reduction in high group) (Figure 1), whereas no changes in HDL-C levels were observed (Figure 1). A diet containing cocoa powder did not affect daily diet intake or body weight (data not shown).

### 3.2. Quantification of Gross Atherosclerotic Lesions

**Figure 2:**
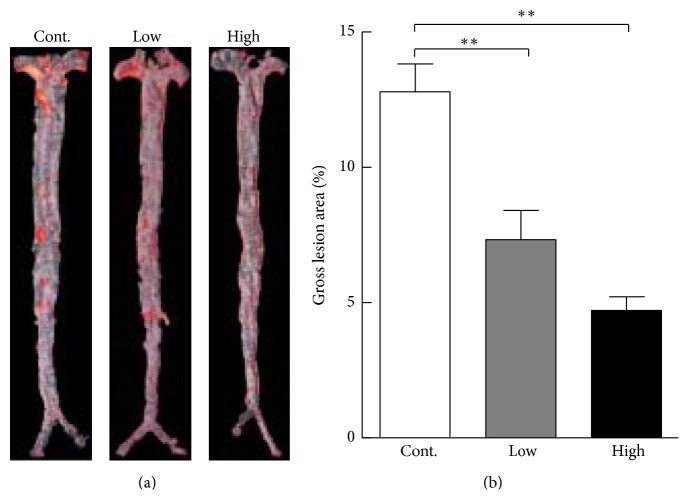
Representative picture of oil red O staining of aortas (a) and quantitative analysis of atherosclerotic lesions (b). Data are expressed as the mean ± SEM. *n* = 12 for each group. ^*∗∗*^ *P* < 0.01 versus control.

Compared with the control group, the* en face* lesion size in the total aorta was significantly reduced, by 42% in the low group and 63% in the high group (Figure 2). Although there was a greater reduction in the high group than in the low group, the difference was not significant.

**Figure 3:**
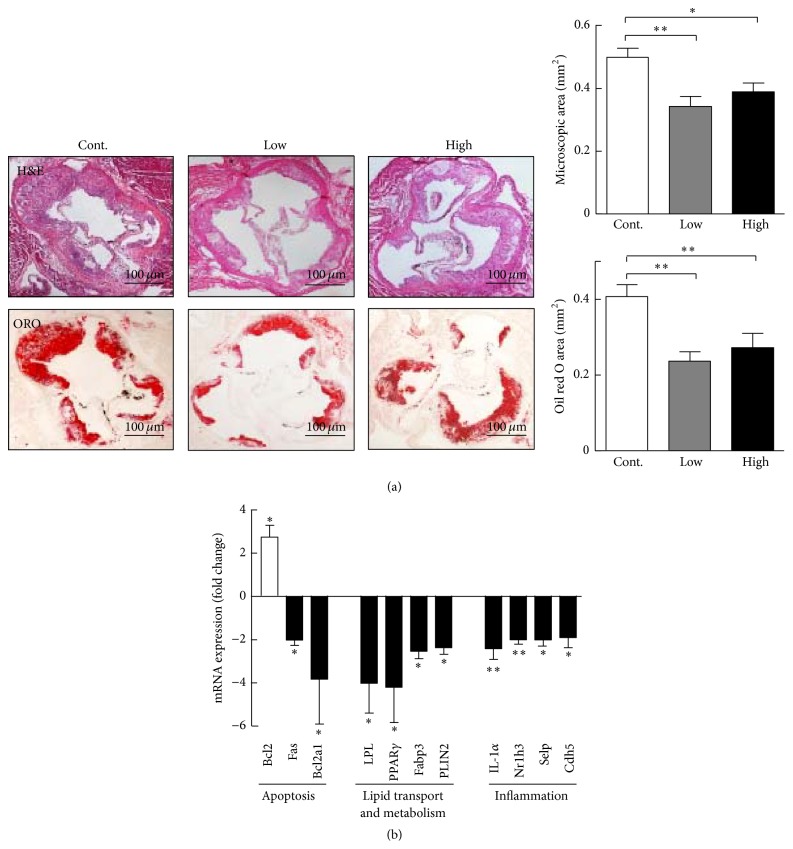
Representative micrographs of atherosclerotic lesions of the aortic root and gene expression changes in the aortas. (a) Aortic root sections are stained with hematoxylin-eosin (H&E) and oil red O (ORO). Quantitative analysis of aortic root lesion areas is shown in the right. *n* = 8 for each group. (b) Gene expression changes in the aortas of the high cocoa powder group relative to the control group. *n* = 3 for each group (pooled samples). Bcl2: B-cell leukemia/lymphoma 2; Fas: tumor necrosis factor receptor superfamily, member 6; Bcl2a1: Bcl2-related protein a1; LPL: lipoprotein lipase; PPAR*γ*: peroxisome proliferator-activated receptor gamma; Fabp3: fatty acid binding protein 3; PLIN2: perilipin 2; IL-1*α*: interleukin-1 alpha; Nr1h3: nuclear receptor subfamily 1, group H, member 3; Selp: selectin P; Cdh5: cadherin 5. Data are expressed as the mean ± SEM. ^*∗*^ *P* < 0.05, ^*∗∗*^ *P* < 0.01 versus control.

Histological examination revealed that aortic root atherosclerotic lesions were also reduced in the cocoa powder groups. Compared with the control group, microscopic intimal lesions in the aortic root of low and high cocoa powder groups were significantly reduced by 30% and 22%, respectively (Figure 3). Accordingly, the lipid area in the lesions stained by oil red O was simultaneously reduced by 47% and 32% in the low and high groups, respectively, while there was no significant difference between the two cocoa powder groups.

### 3.3. Gene Expression in Atherosclerotic Lesions

Because atherosclerotic lesions were significantly reduced in the cocoa powder-fed mice, we attempted to investigate which genes had altered expression levels in the lesions. To accomplish this, we performed a PCR array using samples from the high group and comparing them with the controls. Among 84 genes analyzed, 11 genes were significantly different from the controls (Figure 3). An antiapoptosis gene, B-cell leukemia/lymphoma 2 (Bcl2), was upregulated, while proapoptosis genes such as tumor necrosis factor receptor superfamily member 6 and Bcl-2-related protein a1 were downregulated. Lipid transport and metabolism genes such as peroxisome proliferator-activated receptor gamma (PPAR*γ*), lipoprotein lipase (LPL), fatty acid binding protein 3, and perilipin 2 were decreased compared with the control. Genes which are involved in inflammation, including interleukin-1 beta, nuclear receptor subfamily 1, group H, member 3, selectin P, and cadherin 5, were all reduced in the cocoa powder group compared with the control group.

### 3.4. Antioxidative Effects

**Figure 4:**
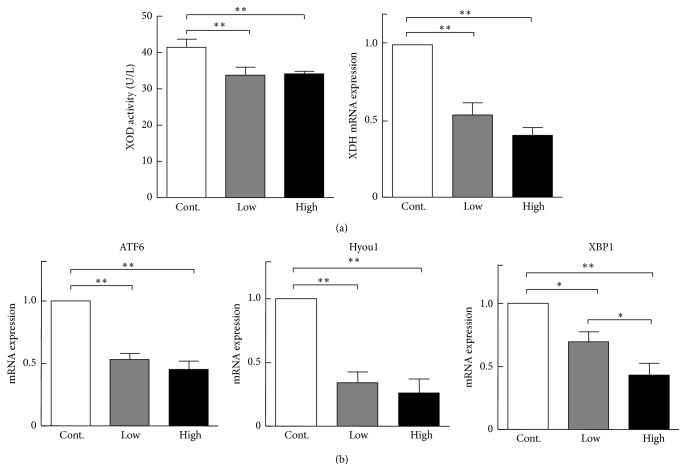
(a) Xanthine oxidase (XOD) protein activity of the plasma and xanthine dehydrogenase (XDH) mRNA expression in the liver. *n* = 3 for each group (pooled samples). (b) RT-PCR results of mRNA expression in the liver. *n* = 6 for each group. Activating transcription factor 6 (ATF6), hypoxia upregulated 1 (Hyou1), and x-box-binding protein 1 (XBP1) mRNA expression levels were analyzed by RT-PCR as described in Section 2. Data are expressed as the mean ± SEM. ^*∗*^ *P* < 0.05, ^*∗∗*^ *P* < 0.01 versus control.

To determine whether cocoa powder had any antioxidative effects, we measured plasma oxidative stress protein levels. As shown in Figure 4, plasma XOD activity was significantly decreased and the precursor of xanthine dehydrogenase (XDH) mRNA expression levels was also reduced significantly in the cocoa powder group. However, superoxidase dismutases, malonyldialdehyde, total nitric oxide synthase, glutathione peroxidase, glutathione *s*-transferase, total antioxidant capacity, and nitric oxide were not significantly changed compared with the control group (Supplementary Figure  1).

### 3.5. Endoplasmic Reticulum Stress in the Liver

**Figure 5:**
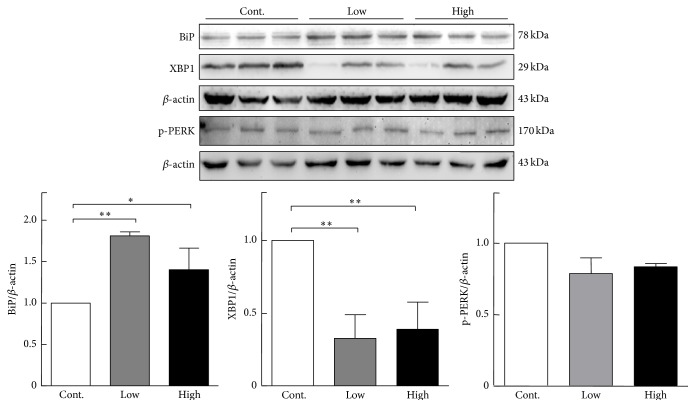
Western blotting analysis of x-box-binding protein 1 (XBP1), binding immunoglobulin protein (BiP), and phosphorylated eukaryotic translation initiation factor 2-kinase 3 (p-PERK) protein expressions in the liver. Data are expressed as the mean ± SEM. *n* = 3 for each group (pooled samples). ^*∗*^ *P* < 0.05, ^*∗∗*^ *P* < 0.01 versus control.

**Figure 6:**
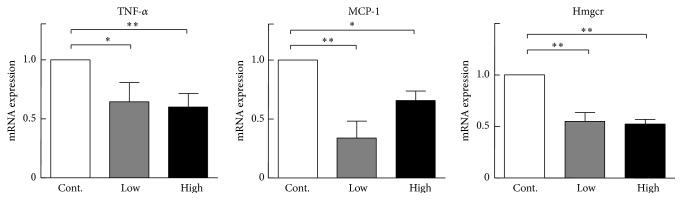
Monocyte chemotactic protein 1 (MCP-1), tumor necrosis factor (TNF*α*), and 3-hydroxy-3-methylglutaryl-coenzyme A reductase (Hmgcr) mRNA expression levels in the liver analyzed by real time PCR. Data are expressed as the mean ± SEM. *n* = 3 for each group (pooled samples). ^*∗*^ *P* < 0.05, ^*∗∗*^ *P* < 0.01 versus control.

Previous studies revealed that chronic endoplasmic reticulum (ER) stress in the liver disturbs lipid metabolism and promotes intracellular cholesterol and fatty acid accumulation [22, 23]. We compared mRNA expression of ER stress-related genes, including activating transcription factor 6, hypoxia upregulated 1, and x-box-binding protein 1 (XBP1), and found that the mRNA expression levels of all these genes were significantly reduced compared with the control group (Figure 4). At the same time, XBP1 protein expression was decreased in the cocoa powder groups. Additionally, binding immunoglobulin protein (BiP) expression was significantly increased compared with the control group and phosphorylation eukaryotic translation initiation factor 2-alpha kinase 3 protein expression levels did not change between three groups (Figure 5). Therefore, intake of cocoa powder inhibits ER stress and maintains the ER homeostasis of the liver. This notion was also supported by the finding that inflammatory reaction genes, tumor necrosis factor alpha and monocyte chemoattractant protein 1, were significantly downregulated in the cocoa powder-fed groups (Figure 6).

**Figure 7:**
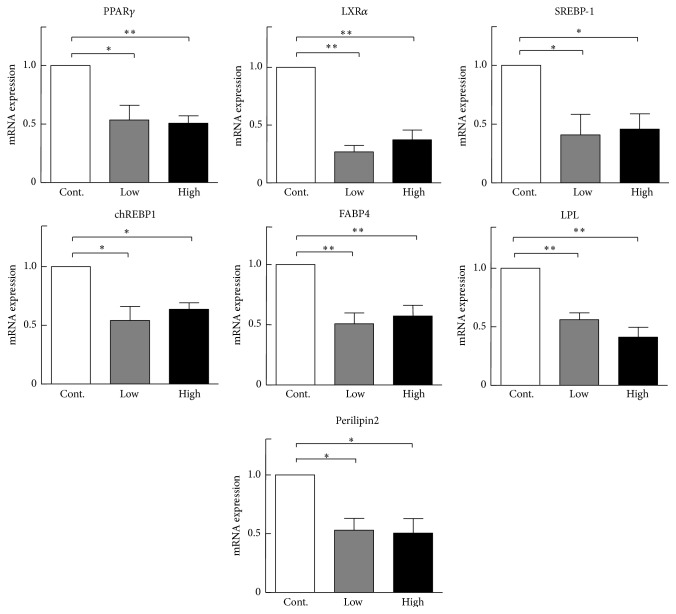
Real time PCR results of lipid and glucose metabolism related genes mRNA expressions levels in the liver. *n* = 3 for each group (pooled samples). Data are expressed as the mean ± SEM. ^*∗*^ *P* < 0.05, ^*∗∗*^ *P* < 0.01 versus control. PPAR*γ*: peroxisome proliferator-activated receptor gamma; LXR*α*: nuclear receptor subfamily 1, group H, member 2; SREBP-1: sterol regulatory element binding transcription factor 1; chREBP1: WS basic-helix-loop-helix leucine zipper protein 1; FABP4: fatty acid binding protein 4; LPL: lipoprotein lipase.

**Figure 8:**
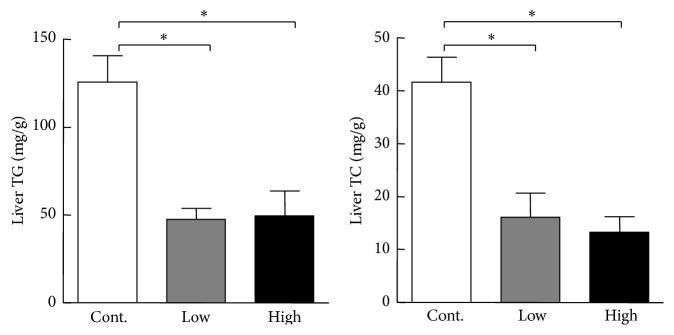
Liver total cholesterol (TC) and triglycerides (TG). Data were analyzed with ANOVA. Data are expressed as the mean ± SEM. *n* = 8 for each group. ^*∗*^ *P* < 0.05 versus control.

To explore the molecular mechanisms responsible for the lower plasma lipids, we compared mRNA expression of lipid metabolism related transcription factors and genes, including carbohydrate response element binding protein, sterol regulatory element binding proteins 1, liver X nuclear receptor alpha variant 1, PPAR*γ*, fatty acid binding protein 4, perilipin 2, and LPL. As shown in Figure 7, these gene expression levels in liver were significantly decreased in cocoa powder groups compared with the control group. In addition, we found that cocoa powder feeding significantly reduced hepatic contents of lipids compared to the control group (Figure 8).

## 4. Discussion

In this study, we demonstrated that intake of 0.2% and 2% of cocoa powder improved hypercholesterolemia and inhibited aortic atherosclerosis in apoE KO mice. These doses can be converted to about 4.2 or 42 g per day used for humans, which is equivalent to the doses reported by others [24, 25].

Both low and high cocoa powder diets had a similar lipid-lowering and antiatherogenic effect, suggesting that the beneficial effects of dietary cocoa powder are not totally dependent upon a high dose. It is likely that maximal effects can be achieved at low doses or high doses cannot be completely absorbed by intestines. Aortic lesions were significantly reduced in the cocoa powder-fed mice, which were characterized by reduced lipid accumulation. Decreased atherosclerotic lesions in the cocoa powder group may be caused by two possible mechanisms. Cocoa powder-containing diets led to the reduction of plasma cholesterol, especially LDL-C levels (i.e., accounting for most atherogenic lipoproteins), thereby inhibiting the development of atherosclerosis through lipid-lowering effects. Indeed, some human studies showed that administration of polyphenol-rich foods such as cocoa powder modulated and decreased LDL-C and increased HDL-C concentrations [26–28]; our study showed that the plasma HDL-C levels also slightly increased. On the other hand, Nicod and coworkers found that polyphenols from cocoa administered at a dietary dose moderately modulate intestinal inflammation but do not increase cholesterol secretion by intestinal cells or enhance HDL functionality [29]. This contention was suggested by the results of a PCR array analysis, which revealed that many atherogenic genes, such as genes related to apoptosis, lipid metabolism, and inflammation, were significantly downregulated in the cocoa powder-fed groups. In this regard, our study demonstrated that the antiapoptosis gene Bcl2 was upregulated. Thorp et al. reported that Bcl2 produced by macrophages plays a protective role against macrophage apoptosis, specifically in advanced atherosclerotic lesions [30]. It is well known that Bcl2 is also involved in cancer and autoimmune diseases [31, 32]. Therefore, it will be interesting to investigate whether cocoa powder has any other beneficial effects on these diseases. It should be pointed out that there is a possibility that upregulation of Bcl2 compensates the downregulation of Bcl2a1 or vice versa induced by cocoa powder.

Namgaladze et al. found that inhibition of PPAR*γ*-dependent gene expression during monocyte differentiation may contribute to AICAR-elicited changes in macrophage phenotypes characterized by reduced inflammatory responses to modified LDLs and saturated fatty acids [33]. Inhibition of PPAR*γ* functions, specifically in the vascular endothelium or smooth muscle cells, also affects the development of cardiovascular disease [34]. Consistent with this finding, our results demonstrated that PPAR*γ* expression was decreased in the lesions of the cocoa powder group. Upregulation of selectin P and cadherin 5 expressed in arterial endothelial cells mediate rapid rolling of leukocytes over vascular cell surfaces during the initial steps in atherogenesis [35].

It is well known that the liver is a central metabolic organ and plays a critical role in fatty acid and cholesterol metabolism [36]. Hepatic ER stress can lead to altered lipid metabolism and hepatic steatosis [37]. Although lipid-lowering effects of cocoa liquid polyphenols were reported in previous studies [38, 39], the molecular mechanisms are largely unclear. In the present study, we revealed that a diet containing cocoa powder led to the inhibition of ER stress in the liver. Because ER stress can lead to hepatic steatosis and altered cholesterol and triglyceride biosynthetic pathways [37], improvement of hepatic ER stress may help explain why a diet containing cocoa powder led to the reduction of plasma cholesterol levels shown in Figure 1.

There are a number of pathophysiological conditions in which ER stress plays a major role. First, glucosamine-induced ER stress can promote lipid accumulation and the activation of inflammatory pathways [40]. Second, ER stress signaling promotes atherogenic lipid accumulation in macrophages [41]. Third, atorvastatin administered in golden hamsters could upregulate BiP protein expression in the aortic root [42], so BiP could exert a protective effect in the early stages of atherosclerosis beyond being a chaperone protein in ER stress. While it remains to be verified in the future, we speculate that a diet containing cocoa powder may also improve ER stress in the lesions of aorta.

In the present study, we found that the hepatic gene expression profile of lipid metabolism was simultaneously changed with an ER stress signaling pathway, which was consistent with the previous study [43]. In the ER lumen, BiP is a glucose-regulated protein that functions as a molecular chaperone [44]; therefore, BiP plays a critical role in maintaining hepatic lipid homeostasis. Our results demonstrated that a diet containing cocoa powder increased BiP protein expression levels associated with reduced hepatic steatosis [45].

In conclusion, dietary cocoa powder improves hypercholesterolemia and inhibits aortic atherosclerosis in apoE KO mice. Although molecular pathways are not fully understood, our study suggests that the improvement of hepatic ER stress may play a major role in the inhibition of atherosclerosis induced by cocoa powder.

## Supplementary Material

The compositions of cocoa powder are shown in Supplementary Table 1.
The detailed mouse Western diet compositions are listed in Supplementary Table 2.
The results of the PCR array are shown in Supplementary Table 3. We attempted to investigate which genes had altered expression levels in the lesions. We performed a PCR array using samples from the high group and comparing them with the controls. n = 3 for each group. Data are expressed as the mean ±SEM.
RT-PCR primer sequences are shown in Supplementary Table 4.

## Abbreviations

Abs: Antibodies
Bcl2: B-cell leukemia/lymphoma 2
BiP: Binding immunoglobulin protein
ER: Endoplasmic reticulum
HDL-C: High density lipoprotein cholesterol
KO: Knockout
LDL-C: Low density lipoprotein cholesterol
LPL: Lipoprotein lipase
PPAR*γ*: Peroxisome proliferator-activated receptor gamma
TC: Total cholesterol
TG: Triglycerides
XBP1: x-box-binding protein 1
XDH: Xanthine dehydrogenase
XOD: Xanthine oxidase.
