# Supplementary material for: Dietary Cocoa Powder Improves Hyperlipidemia and Reduces Atherosclerosis in apoE Deficient Mice through the Inhibition of Hepatic Endoplasmic Reticulum Stress
Source: Mediators Inflamm. 2016 Feb 15;2016:1937572. doi: 10.1155/2016/1937572 (PMC4770140; doi:10.1155/2016/1937572)
Supplement: Supplementary file 1 — The compositions of cocoa powder are shown in Supplementary Table 1. The detailed mouse Western diet compositions are listed in Supplementary Table 2. The results of the PCR array are shown in Supplementary Table 3. We attempted to investigate which genes had altered expression levels in the lesions. We performed a PCR array using samples from the high group and comparing them with the controls. n = 3 for each group. Data are expressed as the mean ±SEM. RT-PCR primer sequences are shown in Supplementary Table 4. [file 1937572.f1.pdf]

Appendices

Supplementary Table 1. Compositions of crude cocoa powder

	**Content (mg/g)**
Macronutrient	
Total fat	100
Total carbohydrate	600
Total protein	200
Polyphenol	
Total monomers	71.0
Epicatechin	4.6
Catechin	2.2
Proanthocyanidins	
Dimer	3.3
Trimer	3.1
Tetramer	3.8
Pentamer	3.2
Hexamer	2.7
Heptamer	2.2
Octamer	2.6
Nonamer	1.5
Decamer	1.7

Foot note: Provided by China General Technology Holding Company.

Supplementary Table 2. Compositions of mouse Western diet

**Nutrient**	**Composition (%)**
Basal mouse feed	78.98
Lard	21
cholesterol	0.15
Energy (kcal/kg)	4510
**Ingredient (%)**	
Protein	15.8
Fat	24.13
Fiber	1.96
Crude ash	2.09
Carbohydrate	42.65
Calcium	0.9
Phosphorus	0.69
Lysine	0.67
Methionine	0.48
Arginine	0.67
Histidine	0.34
Tryptophan	0.14
Phenyl C	1.09
Threonine	0.49
Leucine	1.14
Isoleucine	0.53
Valine	0.62
Magnesium	0.16
Potassium	0.51
Sodium	0.19

Foot note: Provided by Vital River Company

Supplementary Table 3. PCR array results of aortas

Description	Fold change	*P*- value(n=3)
Selectin, endothelial cell	-3.6217	0.132443
Lipoprotein lipase	-3.4742	0.015573
Peroxisome proliferator activated receptor gamma	-3.4263	0.02259
Matrix metallopeptidase 1a (interstitial collagenase)	-3.4027	0.088007
B-cell leukemia/lymphoma 2 related protein A1a	-2.8613	0.036411
Fatty acid binding protein 3, muscle and heart	-2.4794	0.007841
Kinase insert domain protein receptor	-2.4284	0.119178
Perilipin 2	-2.3295	0.003315
Interleukin 1 alpha	-2.308	0.012445
Nuclear factor of kappa light polypeptide gene enhancer in	-2.2294	0.597388
Macrophage scavenger receptor 1	-2.2191	0.171391
Integrin alpha X	-2.214	0.272649
Chemokine (C-C motif) ligand 5	-1.977	0.515914
Fas (TNF receptor superfamily member 6)	-1.977	0.015324
Nuclear receptor subfamily 1, group H, member 3	-1.977	0.043186
Selectin, platelet	-1.9543	0.047703
Chemokine (C-C motif) receptor 2	-1.8747	0.302899
Prostaglandin-endoperoxide synthase 1	-1.8618	0.184362
Cadherin 5	-1.7901	0.046128
Integrin alpha 2	-1.6974	0.156945
Chemokine (C-C motif) receptor 1	-1.6663	0.350763
ATP-binding cassette, sub-family A (ABC1), member 1	-1.6472	0.161991
Tumor necrosis factor, alpha-induced protein 3	-1.6208	0.310106
Lysophospholipase 1	-1.6133	0.121042
Apolipoprotein A-I	-1.5984	0.272968
Integrin beta 2	-1.5911	0.210166
Apolipoprotein E	-1.5801	0.521446
Leukemia inhibitory factor	-1.5801	0.073135
Secreted phosphoprotein 1	-1.5404	0.261536
Interleukin 1 receptor, type II	-1.5263	0.484753
Tumor necrosis factor	-1.5227	0.511643
Chemokine (C-C motif) ligand 2	-1.5192	0.489232
Apolipoprotein B	-1.4406	0.145734
Colony stimulating factor 2 (granulocyte-macrophage)	-1.4406	0.145734
Fibrinogen alpha chain	-1.4406	0.145734
Fibrinogen beta chain	-1.4406	0.145734

Interferon gamma	-1.4406	0.145734
Interleukin 2	-1.4406	0.145734
Interleukin 3	-1.4406	0.145734
Interleukin 5	-1.4406	0.145734
Laminin, alpha 1	-1.4406	0.145734
Thrombospondin 4	-1.4406	0.145734
Retinoid X receptor alpha	-1.4373	0.558118
Vascular endothelial growth factor A	-1.4306	0.444496
Superoxide dismutase 1, soluble	-1.4077	0.058907
Elastin	-1.4044	0.393012
Transforming growth factor, beta 2	-1.4012	0.649983
Matrix metallopeptidase 3	-1.3692	0.284797
Interleukin 4	-1.3629	0.386151
CASP8 and FADD-like apoptosis regulator	-1.2628	0.238132
Transforming growth factor, beta 1	-1.2599	0.219367
BH3 interacting domain death agonist	-1.2368	0.721725
Peroxisome proliferator activated receptor alpha	-1.1975	0.747026
CD44 antigen	-1.1408	0.559023
Intercellular adhesion molecule 1	-1.1408	0.829335
Bcl2-like 1	-1.107	0.439555
Vascular cell adhesion molecule 1	-1.0968	0.873811
Collagen, type III, alpha 1	-1.0943	0.467957
Angiotensin I converting enzyme (peptidyl-dipeptidase A) 1	-1.0743	0.80899
Chemokine (C-X-C motif) ligand 1	-1.0473	0.671943
Fibroblast growth factor 2	-1.007	0.894246
Actin, beta	1	0
Connective tissue growth factor	1.0401	0.982886
Endoglin	1.0595	0.911608
Selectin, platelet (p-selectin) ligand	1.0595	0.777916
Heparin-binding EGF-like growth factor	1.0792	0.767953
Integrin alpha 5 (fibronectin receptor alpha)	1.0943	0.851573
Interleukin 1 beta	1.0994	0.922189
Neuropeptide Y	1.1173	0.622566
Peroxisome proliferator activator receptor delta	1.1303	0.734718
Platelet derived growth factor receptor, beta polypeptide	1.1892	0.27901
Selectin, lymphocyte	1.1892	0.424324
Low density lipoprotein receptor	1.192	0.371272
Platelet derived growth factor, alpha	1.2834	0.464115
Von Willebrand factor homolog	1.2834	0.557175

Bcl2-associated X protein	1.2894	0.42552
Serine (or cysteine) peptidase inhibitor, clade B, member 2	1.2924	0.250784
Kruppel-like factor 2 (lung)	1.3472	0.265187
Platelet derived growth factor, B polypeptide	1.4109	0.282982
Fibronectin 1	1.434	0.32549
Interleukin 1 receptor, type I	1.4439	0.170929
Baculoviral IAP repeat-containing 3	1.4914	0.305429
Serine (or cysteine) peptidase inhibitor, clade E, member 1	1.5192	0.39205
Tenascin C	1.6818	0.291151
B-cell leukemia/lymphoma 2	2.639	0.016784

Foot note: High dose cocoa powder group vs. Control group

Supplementary Table 4. Primers used for Real time RT PCR

Genes	Primers (5’ to 3’)
SREBP1-F	CCGAGATGTGCGAACTGGA
SREBP1-R	GAAGTCACTGTCTTGGTTGTTGATG
chREBP -F	CAGCATCGATCCGACACTCA
chREBP -R	ATCTTGTCCCGGCATAGCAAC
PPARγ-F	GGAGCCTAAGTTTGAGTTTGCTGTG
PPARγ-R	TGCAGCAGGTTGTCTTGGATG
LXRα-F	CTGGAGACGTCACGGAGGTACA
LXRα-R	TGATGGCAATGAGCAGAGCA
Perilipin2 -F	GAGCTGGAGATGGAAGCAAAA
Perilipin2 -R	GTGATAAGCCCGAGAGCAGAG
LPL-F	GTCCCCTGGTCGAAGCATT
LPL-R	TCCACATCTCCAAGTCCTCTCTC
XDH-F	ATCTGGAGACCCACTGCACC
XDH-R	TGTGCTCACGAAGAGCTCCAT
Fabp4-F	AGCGTAAATGGGGATTTGGTC
Fabp4-R	GCTCTTCACCTTCCTGTCGTCT
Hyou1-F	TATCCAGCCTGTTTGGAGGTG
Hyou1-R	GCTCTGCAGGCTCATCCTTG
ATF6-F	AGTCCCAAGTCCAAAGCGAAGA
ATF6-R	CTGATTGGCAGGGCTCACACTA
XBP1-F	CAGCAAGTGGTGGATTTGGAAG
XBP1-R	TCTTAACTCCTGGTTCTCAACCAC
TNF-α-F	TATGGCCCAGACCCTCACA
TNF-α-R	GGAGTAGACAAGGTACAACCCATC
MCP1-F	AGCAGCAGGTGTCCCAAAGA
MCP1-R	GTGCTGAAGACCTTAGGGCAGA
β-actin-F	TGATCTCCTTCTGCATCCTGTC
β-actin-R	GCGGCATCCACGAAACTAC

Figure S1.

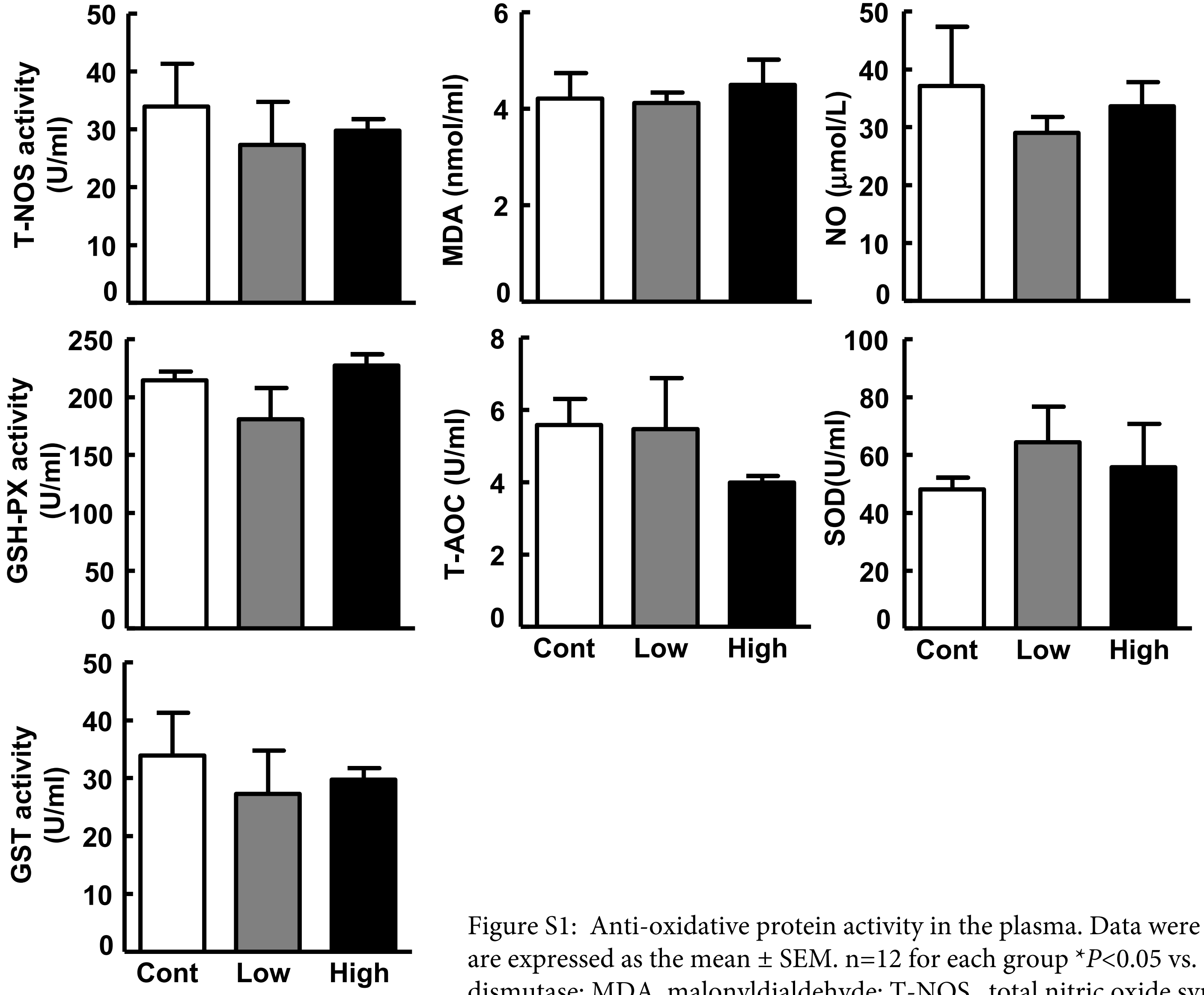

Figure S1: Anti-oxidative protein activity in the plasma. Data were analyzed with an ANOVA. Data are expressed as the mean ± SEM. n=12 for each group **P*<0.05 vs. control. SOD, Superoxidase dismutase; MDA, malonyldialdehyde; T-NOS, total nitric oxide synthase; GSH-PX, glutathione peroxidase; GSH-ST, glutathione s-transferase; T-AOC, total antioxidant capacity; XOD, xanthine oxidase; NO, nitric oxide.
